# Supplementary material for: Egyptian Fruit Bats (Rousettus aegyptiacus) Were Resistant to Experimental Inoculation with Avian-Origin Influenza A Virus of Subtype H9N2, But Are Susceptible to Experimental Infection with Bat-Borne H9N2 Virus
Source: Viruses. 2021 Apr 14;13(4):672. doi: 10.3390/v13040672 (PMC8070959; doi:10.3390/v13040672)
Supplement: Supplementary file 1 [file viruses-13-00672-s001.pdf]

Supplementary Data

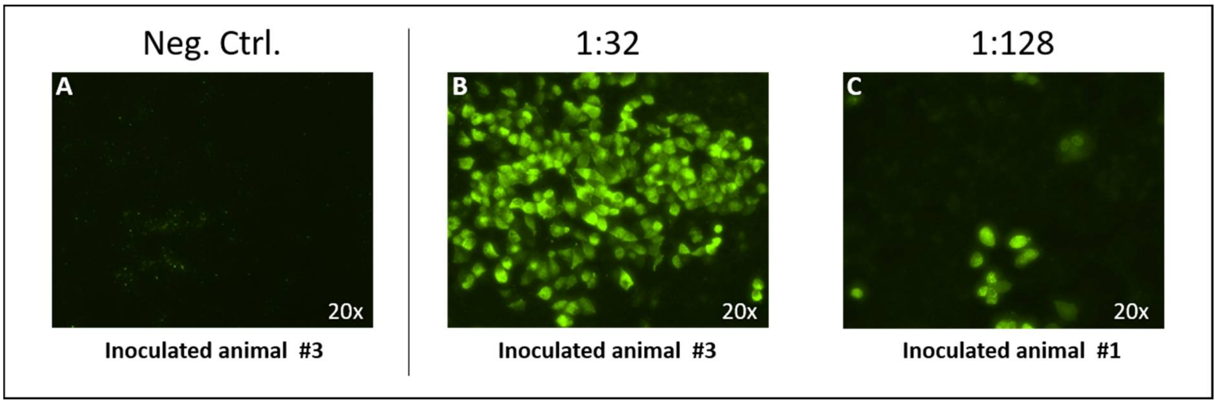

Supplementary Figure S1: Immunofluorescence assay of animal sera showing sero-reactivity after infection of Egyptian fruit bats with A/bat/Egypt/381OP/2017 (H9N2). Cells were seeded in a 96-well plate and grown for 24 hours. Afterwards, cells were infected with A/bat/Egypt/381OP/2017 (H9N2) for 24 hours, followed by fixation with 4% paraformaldehyde (PFA) and permeabilization with 0.5% Triton-X-100 in PBS. Finally, cells were incubated for 1 h with the respective animal sera in a dilution series of 1:10 – 1:256, as well as with a negative serum sample of each respective animal taken before start of the experiment. Pictures were taken at a 20x magnification **A** Fixed and permeabilized infected cells were incubated for 1 h with negative serum of the respective animal taken before start of the experiment. **B** Fixed and permeabilized infected cells were incubated for 1 h with a 1:32 dilution of serum taken from the respective inoculated animal #3 at 21 dpi **C** Fixed and permeabilized infected cells were incubated for 1 h with a 1:128 dilution of serum taken from the respective inoculated animal #1 at 21 dpi.

Supplementary Table S1: S/P-values of the NP-ELISA with sera collected from animals that were infected with A/bat/Egypt/381OP/2017 (H9N2)

	**S/P-value d0**	**S/P-value d7**	**S/P-value d21**
**Contact animal #1**	106,4039871		105,6568482
**Contact animal #2**	106,8042283		99,92884341
**Contact animal #3**	103,9224447		101,1295883
**Contact animal #4**	104,9719806		99,17281922
**Contact animal #5**	110,0951702	101,2630056	
**Contact animal #6**	109,9261799	98,55021231	
**Inoculated animal #1**	105,8169531		21,02641578
**Inoculated animal #2**	105,3633365		45,21924945
**Inoculated animal #3**	107,7292534		13,25269088
**Inoculated animal #4**	107,4268458		53,20644148
**Inoculated animal #5**	107,1422194	70,62172262	
**Inoculated animal #6**	108,6631637	82,27341541	

≤45% positive; 45% - 50% questionable; ≥50 % negative
